# Who are vaccine champions and what implementation strategies do they use to improve adolescent HPV vaccination? Findings from a national survey of primary care professionals

**DOI:** 10.1186/s43058-024-00557-0

**Published:** 2024-03-22

**Authors:** Micaela K. Brewington, Tara L. Queen, Jennifer Heisler-MacKinnon, William A. Calo, Sandra Weaver, Chris Barry, Wei Yi Kong, Kathryn L. Kennedy, Christopher M. Shea, Melissa B. Gilkey

**Affiliations:** 1 Department of Health Behavior, Gillings School of Global Public Health, University of North Carolina, Chapel Hill, NC USA; 2 Department of Public Health Sciences, Penn State College of Medicine, Hershey, PA USA; 3 UNC Family Medicine and Pediatrics, UNC Health, Chapel Hill, NC USA; 4 JMA Pediatrics, Raleigh, NC USA; 5 Department of Health Policy and Management, Gillings School of Global Public Health, University of North Carolina, Chapel Hill, NC USA; 6 Lineberger Comprehensive Cancer Center, University of North Carolina, Chapel Hill, NC USA

**Keywords:** Champions, HPV vaccines, Immunizations, Primary care, Implementation strategy, Evidence-based practice, Adolescent health services, Health communication

## Abstract

**Background:**

Implementation science researchers often cite clinical champions as critical to overcoming organizational resistance and other barriers to the implementation of evidence-based health services, yet relatively little is known about who champions are or how they effect change. To inform future efforts to identify and engage champions to support HPV vaccination, we sought to describe the key characteristics and strategies of vaccine champions working in adolescent primary care.

**Methods:**

In 2022, we conducted a national survey with a web-based panel of 2527 primary care professionals (PCPs) with a role in adolescent HPV vaccination (57% response rate). Our sample consisted of pediatricians (26%), family medicine physicians (22%), advanced practice providers (24%), and nursing staff (28%). Our survey assessed PCPs’ experience with vaccine champions, defined as health care professionals “known for helping their colleagues improve vaccination rates.”

**Results:**

Overall, 85% of PCPs reported currently working with one or more vaccine champions. Among these 2144 PCPs, most identified the champion with whom they worked most closely as being a physician (40%) or nurse (40%). Almost all identified champions worked to improve vaccination rates for vaccines in general (45%) or HPV vaccine specifically (49%). PCPs commonly reported that champion implementation strategies included sharing information (79%), encouragement (62%), and vaccination data (59%) with colleagues, but less than half reported that champions led quality improvement projects (39%). Most PCPs perceived their closest champion as being moderately to extremely effective at improving vaccination rates (91%). PCPs who did versus did not work with champions more often recommended HPV vaccination at the earliest opportunity of ages 9–10 rather than later ages (44% vs. 33%, *p* < 0.001).

**Conclusions:**

Findings of our national study suggest that vaccine champions are common in adolescent primary care, but only a minority lead quality improvement projects. Interventionists seeking to identify champions to improve HPV vaccination rates can expect to find them among both physicians and nurses, but should be prepared to offer support to more fully engage them in implementing interventions.

**Supplementary Information:**

The online version contains supplementary material available at 10.1186/s43058-024-00557-0.

Contributions to the literature

- We surveyed 2527 US primary care professionals (PCPs) to describe key characteristics and strategies of vaccine champions in adolescent primary care.
- Most PCPs (85%) worked with vaccine champions, with similar proportions identifying a physician or nurse as their closest champion.
- PCPs commonly reported that champion provided information (79%), encouragement (62%), and vaccination data (59%) to colleagues, but less than half reported champions led quality improvement projects (39%).
- Working with a champion correlated with more positive HPV vaccine recommendation practices and clinic performance perceptions.
- Findings suggest vaccine champions are common, but may need more support to be quality improvement leaders.

## Introduction

Implementation science research emphasizes the importance of clinical champions in scaling up the implementation of evidence-based health services. According to the Expert Recommendations for Implementing Change (ERIC), champions are “individuals who dedicate themselves to supporting, marketing, and driving through an implementation, overcoming indifference or resistance that the intervention may provoke in an organization” [1]. Champions are characterized by their persistence, enthusiasm, and conviction in pushing implementations forward, even when it means putting their reputations on the line [2]. They differ from related concepts, such as “opinion leaders,” who more passively exert an influence on the flow of information within networks [3]. In this way, champions constitute an implementation strategy in and of themselves [1], while also having robust potential to effectively deliver training and other support to improve the provision of evidence-based services within clinics and larger health care systems. Perhaps not surprisingly, interventions in clinical settings commonly feature a champion component [2, 4–6].

Human papillomavirus (HPV) vaccination is a useful case study for investigating the role of champions. Widespread HPV vaccination could prevent over 90% of the nearly 36,500 HPV cancers diagnosed in the United States each year [7]. Unfortunately, despite national recommendations for adolescents to receive the two-dose HPV vaccine series between ages 9 and 12, only 50% of 13-year-olds were fully vaccinated in 2021, with consistently lower coverage in rural areas [8, 9]. Importantly, younger age at initiation of the HPV vaccine series is associated with higher rates of on-time series completion [10]. The reasons for low uptake are complex, but one key factor is primary care professionals’ (PCPs’) infrequent and ineffective recommendation of HPV vaccination [11–13]. Evidence-based implementation strategies that combine provider communication training, assessment and feedback, and other techniques are emerging to improve HPV vaccination within health care settings [14–16]. Given their role as change agents, training champions to use these implementation strategies could help address challenges with scaling routine HPV vaccination across health care systems.

Despite the implementation research literature consistently emphasizes the critical importance of champions, relatively little work has provided insight into how to identify and engage champions to best meet implementation needs. For example, no prior studies have examined the extent to which champion relationships are characterized by homophily in clinical role such that physicians look to physicians as champions, while nurses look to other nurses. Further, prior work has not specifically explored champions in the context of HPV vaccination, though the presence of a champion has been positively associated with HPV vaccination performance in primary care [17]. Thus, we conducted a national survey of adolescent PCPs to evaluate how common vaccine champions are, their roles and attributes, and implementation strategies they use to promote adolescent vaccination, including HPV vaccination. Our findings may guide future efforts to identify, engage, and train champions to deliver evidence-based interventions to support HPV vaccination within clinical settings.

## Methods

### Participants and procedures

We conducted a web-based survey in May–July 2022 to assess PCPs’ perceptions of and experiences working with vaccine champions in adolescent primary care. Eligible PCPs were physicians, advanced practice providers (i.e., physician assistants and advanced practice nurses), and nursing staff (registered nurses, licensed practical/vocational nurses, medical assistants, and certified nursing assistants). Additionally, eligible respondents (1) were certified to practice in the US; (2) worked in pediatrics or family medicine and general practice (hereafter “family medicine”); and (3) had one or more roles in HPV vaccination for children ages 9–12. Roles in HPV vaccination were specified as assessing children’s vaccination status, notifying parents when children are due for the vaccine, recommending the vaccine, addressing parent questions and concerns, or administering the vaccine.

We contracted with a survey company, WebMD Market Research, to recruit PCPs through the Medscape Network, which provides web-based information, continuing education, and research participation opportunities to the medical community. About 60% of US physicians are members of the network, and Medscape verifies physicians’ and advanced practice providers’ licenses upon registration. In the pre-recruitment phase, the survey company constructed a survey panel by emailing members with the appropriate medical training (i.e., physicians, advanced practice providers, and nursing staff) to assess their interest in survey participation and to filter out inactive members. Members who responded affirmatively were eligible to join the study.

In the recruitment phase, the survey company emailed 6278 panel members a link to the web-based survey, followed by up to four reminders for members who did not respond. We used quotas to ensure balance in our sample by medical training. More specifically, we aimed to include roughly equal proportions of pediatricians, family physicians, advanced practice providers, and nursing staff. Because of rural-urban disparities in HPV vaccination, we oversampled PCPs practicing in clinics located in rural counties, as defined by USDA Rural-Urban Continuum Codes (RUCC) 4-9 [9, 18].

Respondents who clicked the survey link began by completing a 4-item screener that ensured they met eligibility criteria (Supplemental Table 1). A total of 2527 PCPs were eligible, provided informed consent, and completed the survey, yielding a response rate of 57% (Response rate 3, [19]). Respondents in our sample compared favorably to those in the general population on key demographic characteristics (Supplemental Table 2). The median completion time for our survey was 19 min, and respondents received an incentive of up to $45 depending on local market rates for survey research participation. The University of North Carolina Institutional Review Board approved the study protocol. We used the Strengthening the Reporting of Observational Studies in Epidemiology (STROBE) cross-sectional study guidelines to develop this manuscript [20].

### Measures

Our survey began by defining “vaccine champion” with the following statement:

Some health care professionals are known for helping their colleagues improve vaccination rates. They are passionate about sharing vaccine-related information, data, tools, and encouragement with others in their clinic. We will call them vaccine champions.

Respondents next reported how many champions they currently work with, using nine response options that ranged from “0 champions” to “8 or more champions.” This item instructed respondents to “Consider anyone who goes above and beyond to help you or others in your clinic improve vaccination rates. You can count physicians, nursing staff, administrators, quality improvement staff, and others” (Supplemental Table 1). We re-categorized responses as working with any vaccine champion (≥ 1 champion) versus none (0 champions) in order to compare these two groups on their characteristics to understand which PCPs may lack this resource.

For PCPs who worked with any champions, the survey used seven closed-ended items to characterize the champion with whom the respondent worked most closely. One of these items assessed how closely the respondent worked with the champion, using a 5-point response scale to rate the tie as “extremely” to “not at all” close. Another three items used prespecified lists to assess the champion’s medical training, clinical role, and how their role as a vaccine champion is recognized in the clinic. The remaining three items used prespecified lists to assess strategies the champion uses to improve vaccination rates, the kind of vaccination rates they work to improve, and the qualities that best describe them.

We used three survey items to assess champion effectiveness. One of these items assessed perceived effectiveness; respondents used a 5-point response scale to rate their closest champion on effectiveness at improving vaccination rates (“Not at all effective” [1] to “extremely effective” [5]). One closed-ended item assessed respondents’ own HPV vaccine-related behavior in terms of the age at which they begin routinely recommending HPV vaccination for their patients; we recategorized response as 9–10 years, 11–12 years, ≥ 13 years, or never. We used a skip pattern to offer this item only to respondents who indicated having a role in HPV vaccine recommendations. One closed-ended item assessed respondents’ perception of their own clinic’s HPV vaccination rates in terms of whether those rates were at or above their state’s average versus below it. For this item, the survey displayed their state’s HPV vaccination rates for their reference.

Our survey assessed the characteristics of respondents and the clinics in which they worked. Demographic and professional characteristics included PCPs’ gender, race/ethnicity, number of years in practice, and number of patients, ages 9-12, that they see in a typical week. Clinical characteristics included practice type and whether the clinic was part of a healthcare system or network. Two items were collected the county and state of the PCP’s primary clinic, which we used to categorize clinics as rural (RUCC 4–9) or nonrural (RUCC 1-3) [18].

Prior to fielding our survey, we cognitively tested subsets of survey items with 16 PCPs recruited for that purpose, as well as with seven additional PCPs who made up our study’s clinical advisory board. These PCPs included physicians, advance practice providers, nurses, and medical assistants who worked in primary care and were not survey participants. Cognitive interviews used “think aloud” activities to assess whether participants interpreted concepts such as “vaccine champion” as intended by the research team. Their feedback helped the study team to define champions in a way that better distinguished the role of “helping colleagues improve” from more general vaccine promotion with patients and their families. PCPs also provided feedback on the comprehensibility of survey items, including the appropriateness of item wording and response options [21].

### Statistical analysis

We used bivariate logistic regression to identify correlates of working with any vaccine champions, modeling the outcome as yes (“≥1 champion”) versus no (“0 champions”). We then entered statistically significant correlates into a multivariable model. We used chi-square tests to assess the association between working with any vaccine champions and each of two effectiveness measures: the age at which respondents delivered routine HPV vaccine recommendations and respondents’ perception of their clinics’ HPV vaccination rates. We conducted analyses using SAS (v 9.4). Statistical tests were two-tailed with a critical alpha of .05.

## Results

### Participant characteristics

Our sample was comprised of pediatricians (26%), family physicians (22%), advanced practice providers (24%), and nursing staff (28%, Table 1). Over two-thirds of PCPs were women (72%). Most respondents identified as White (66%), Asian (14%), Black (5%), or Hispanic (4%). Our sample included PCPs with a range of practice experience, from low (0–9 years, 37%) to medium (10–19 years, 29%) to high (≥ 20 years, 33%).

**Table 1:** Sample characteristics (*n*=2527)

	*n*	(%)
Respondent characteristics		
Training		
Pediatrician	666	(26.4)
Family physician	557	(22.0)
Advanced practice provider^a^	603	(23.9)
Nursing staff^b^	701	(27.7)
Gender		
Woman	1810	(71.6)
Man	637	(25.2)
Another gender/prefer not to say^c^	80	(3.2)
Race and ethnicity		
Asian	356	(14.1)
Black	123	(4.9)
Hispanic	100	(4.0)
White	1664	(65.9)
Multiple races or ethnicities	94	(3.7)
Another race/prefer not to say^d^	190	(7.5)
Years in practice		
0–9	950	(37.6)
10–19	740	(29.3)
≥20	837	(33.1)
Patients age 9–12 seen in typical week		
≤9	730	(28.9)
10–24	1000	(39.6)
≥25	797	(31.5)
Clinic or practice characteristics		
Practice type		
Solo or group	1534	(60.7)
Other^e^	993	(39.3)
Healthcare system membership		
No	963	(38.1)
Yes^f^	1564	(61.9)
Rurality		
Non-rural	2295	(90.8)
Rural	232	(9.2)
Region		
Northeast	505	(20.0)
Midwest	576	(22.8)
South	841	(33.3)
West	605	(23.9)

^a^ Includes physician assistants (*n*=198) and advance practice nurses (*n*=405), including nurse practitioners and clinical nurse specialist

^b^ Includes registered nurses (*n*=542), licensed practical or vocational nurses (*n*=64), certified nursing assistants (*n*=11), and medical assistants (*n*=84)

^c^ Includes nonbinary or another gender (*n*=10) and prefer not to say (*n*=70)

^d^ Includes American Indian or Alaska Natives (*n*=10), Middle Eastern or North Africans (*n*=19), Native Hawaiian or Pacific Islanders (*n*=4), other race or ethnicity (*n*=13), and prefer not to say (*n*=144)

^e^ Includes hospital- and university-affiliated clinic (*n*=512), Federally Qualified Health Center (*n*=272), state or local public health department (*n*=37), local, community, or non-profit clinic (*n*=116), and other (*n*=56)

^f^ Defined as “part of a healthcare system or network.” Includes systems of 1–4 clinics (*n*=423) and 5 or more clinics (*n*=1141)

### Correlates of working with a vaccine champion

Overall, 85% of respondents reported that they currently work with one or more vaccine champions, with 3 champions being the median response for the sample overall. In the multivariable analysis, working with a champion was more common among family physicians, advanced practice providers, and nursing staff compared to pediatricians (81%, 87%, and 90% vs. 80%, *p<*.05, Table 2). Working with a champion was also more common among PCPs who saw medium and high versus lower volumes of 9- to 12-year-old patients (86% and 90% vs. 78%, *p<*.05), as well as among those who did versus did not work in a healthcare system (86% vs.82%, *p<*.05). Working with a champion was less common among PCPs working in the South and the West versus the Northeast (84% and 83% vs. 88%, *p<*.05). Although PCP female gender correlated with working with a champion in bivariate analyses, this association did not retain statistical significance in the multivariable model.

**Table 2:** Correlates of working with a vaccine champion (*n*=2527)

	PCPs who work with ≥1 champion/total PCPs in category (%)		Bivariate			Multivariable		
			OR	(95% CI)	*p*	OR	(95% CI)	*p*
Respondent characteristics								
Training								
Pediatrician	532/666	(79.9)	1	Reference		1	Reference	
Family physician	451/557	(81.0)	1.07	(.81–1.42)	.63	1.55	(1.14–2.11)	.01
Advanced practice provider	527/603	(87.4)	1.75	(1.29–2.37)	<.001	2.15	(1.56–2.98)	<.001
Nursing staff	634/701	(90.4)	2.38	(1.74–3.27)	<.001	2.46	(1.74–3.38)	<.001
Gender								
Woman	1559/1810	(86.1)	1.43	(1.12–1.81)	<.01	1.15	(0.88–1.49)	.31
Man	518/637	(81.3)	1	Reference		1	Reference	
Another gender/prefer not to say	67/80	(83.8)	1.18	(0.63–2.22)	0.60	0.93	(0.49–1.76)	.82
Race								
Asian	301/356	(84.6)	0.97	(0.71–1.32)	0.99			
Black	101/123	(82.1)	0.81	(0.50–1.31)	0.41			
Hispanic	85/100	(85.0)	1.00	(0.57–1.76)	0.88			
White	1414/1664	(85.0)	1	Reference				
Multiple races or ethnicities	79/94	(84.0)	0.93	(0.53–1.64)	0.87			
Another race/prefer not to say	164/190	(86.3)	1.12	(0.72–1.72)	0.46			
Years in practice								
0–9	803/950	(84.5)	1	Reference				
10–19	631/740	(85.3)	1.06	(.81–1.39)	.67			
≥20	710/837	(84.8)	1.02	(.79–1.33)	.86			
Patients age 9–12 seen in typical week								
≤9	567/730	(77.7)	1	Reference		1	Reference	
10–24	859/1000	(85.9)	1.75	(1.37–2.25)	<.001	1.98	(1.52–2.58)	<.001
≥25	718/797	(90.1)	2.61	(1.95–3.49)	<.001	2.83	(2.07–3.88)	<.001
Clinic or practice characteristics								
Practice type								
Solo or group	1295/1534	(84.4)	1	Reference				
Other	849/993	(85.5)	1.09	(0.87–1.36)	.46			
Part of a healthcare system								
No	792/963	(82.2)	1	Reference		1	Reference	
Yes	1352/1564	(86.4)	1.38	(1.11–1.72)	<.01	1.40	(1.12–1.76)	<.01
Rurality								
Non-rural	1951/2295	(85.0)	1	Reference				
Rural	193/232	(83.2)	0.87	(0.61–1.25)	.46			
Region								
Northeast	445/505	(88.1)	1	Reference		1	Reference	
Midwest	493/576	(85.6)	0.80	(0.56–1.14)	0.22	0.76	(0.53–1.09)	.14
South	707/841	(84.1)	0.71	(0.51–0.99)	0.04	0.70	(0.50–0.98)	.04
West	499/605	(82.5)	0.64	(0.45–0.89)	0.01	0.68	(0.48–0.96)	.03

*PCP* primary care professional, *OR* odds ratio, *CI* confidence interval

### Champion attributes

PCPs who worked with at least one champion reported on attributes of the champion with whom they worked mostly closely (Table 3). Among these 2,144 PCPs, most reported that they worked very (41%) to extremely (19%) closely with this champion versus moderately closely or less. Champions identified by PCPs most often worked as patient care team members (80%), and about half of PCPs reported that their closest champions’ role was recognized in their job description (38%) and/or job title (19%).

**Table 3:** Champion attributes and strategies (*n*=2144)

	*n*	(%)
Closeness of tie		
Extremely	416	(19.4)
Very	873	(40.7)
Moderately	653	(30.5)
Slightly or not at all	202	(9.4)
Clinical roles^a^		
Patient care team member	1711	(79.8)
Vaccine stock manager	528	(24.6)
Clinic manager	317	(14.8)
Other administrator	267	(12.5)
Quality improvement coordinator	355	(16.6)
Champion roles		
Part of formal job description	813	(37.9)
Part of job title	413	(19.3)
Neither	1074	(50.1)
Training		
Physician	865	(40.3)
Advanced practice provider	374	(17.4)
Nursing staff	863	(40.3)
None of these	42	(2.0)
Qualities^a^		
Knowledgeable about vaccines	1948	(90.9)
Trusted by patients and families	1799	(83.9)
Effective communicator	1788	(83.4)
Knowledgeable about clinic	1657	(77.3)
Highly respected by colleagues	1586	(74.0)
Strategies^a^		
Communicates effectively with patients and families	1828	(85.3)
Shares information with colleagues	1686	(78.6)
Encourages colleagues to improve	1329	(62.0)
Shares data on vaccination rates	1258	(58.6)
Leads quality improvement projects	838	(39.1)
Targeted vaccinations		
All	974	(45.4)
Select^a^		
HPV	1052	(49.1)
Seasonal influenza	1009	(47.1)
COVID-19	770	(35.9)
Other pediatric vaccinations	930	(43.4)
Other adult vaccinations	261	(12.2)
Perceived effectiveness at improving vaccination rates		
Extremely effective	192	(9.0)
Very effective	868	(40.5)
Moderately effective	879	(40.9)
Slightly or not at all effective	205	(9.6)

^a^ Item allowed multiple selections

**Fig. 1:**
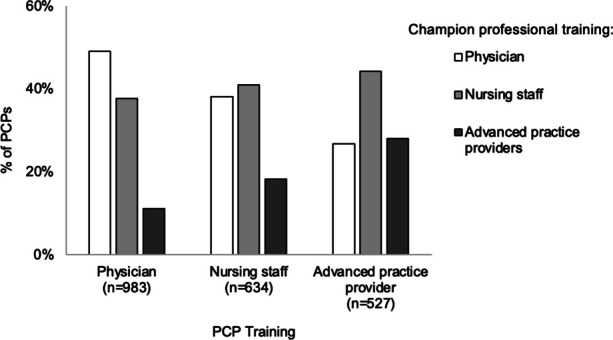
Training of PCPs’ closest champion (*n*=2144)

Over one-third of PCPs identified their closest champion as a physician (40%) or nursing staff member (40%), while the remaining one-fifth identified an advance practice provider (17%) or other role (2%, Table 3). With respect to homophily, physician respondents (*n*=983) identified similar proportions of physicians and non-physicians as their closest champion (49% vs. 49%, Fig. 1). Less than half of nursing staff respondents (*n*=634) identified another nurse as their closest champion, compared to over half who identified a non-nurse (41% vs. 56%). Only about one-fourth (28%) of advanced practice providers (n=527) identified another advance practice provider as their closest champions, compared to almost three-quarters who identified a physician or nurse (28% vs 71%).

Most PCPs described their closest champion as being knowledgeable about vaccines (91%), trusted by patients and families (84%), an effective communicator (83%), knowledgeable about their clinic (77%), and highly respected by colleagues (74%). Regarding the strategies used to improve vaccination rates, PCPs most often reported that their closest champion communicates effectively with patients and families (85%), shares information with colleagues (79%), encourages colleagues to improve (62%), and shares data on vaccination rates (59%); only a minority of PCPs reported their closest champion leads quality improvement projects (39%). Nearly half of respondents reported that their closest champion works to improve vaccination rates for all vaccines (45%) versus select vaccines such as HPV (49%), seasonal influenza (47%), or COVID-19 vaccines (36%).

### Champion effectiveness

**Fig. 2:**
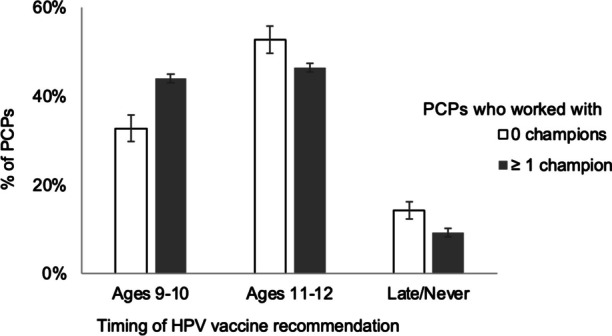
Timing of PCPs’ HPV vaccine recommendations (*n*=2294). Bars show standard error

**Fig. 3:**
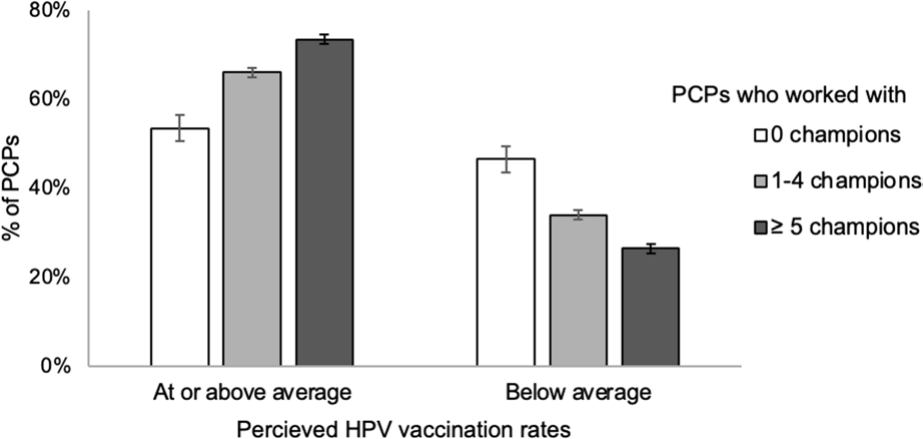
PCPs’ perceptions of their clinic’s HPV vaccination rates (*n*=2527). Bars show standard error

Most PCPs perceived their closest vaccine champion to be moderately to extremely effective at improving vaccination rates (91%, Table 3). Furthermore, working with a vaccine champion was associated with HPV vaccine recommendation timing (*χ*^2^ = 18.07, *p* < .001, Fig. 2). More specifically, among the 2294 PCPs who reported recommending HPV vaccine, those who did versus did not work with champions more often reported beginning routine HPV vaccine recommendations at the earliest opportunity of ages 9-10 (44% vs. 33%) and less often reported recommending HPV vaccine later or never. Finally, working with vaccine champions was associated with higher perceived HPV vaccination rates (*χ*^2^ = 31.78, *p* < .001, Fig. 3); PCPs working with champions more often perceived that their clinic’s vaccination rates were at or above their state’s average (68%) compared to those who do not work with a champion (54%).

## Discussion

Our study is among the first to detail the roles and characteristics of vaccine champions. Our findings suggest that such champions are common in adolescent primary care, with over four-fifths of PCPs in our national sample reporting that they currently work with one or more champion. Most PCPs characterized the tie to their closest champion as very or extremely close and endorsed that person as having broadly positive qualities. Common champion implementation strategies included encouraging colleagues and sharing information and vaccination data, although only a minority of PCPs reported that champions led quality improvement projects. In this way, champions appear to be a pervasive, but potentially underused resource. Champions may require additional training and support if they are to engage their colleagues in more formal initiatives to improve vaccination rates [6]. Future research should explore barriers and facilitators to champions conducting such work, including champion motivation and willingness, as well as opportunities to support them in selecting the most appropriate implementation strategies for meeting their goals.

In addition to underscoring the importance of vaccine champions for improving vaccination rates in general, our study suggests that champions influence HPV vaccination specifically. PCPs perceived champions as effective in improving vaccination rates and most often identified HPV vaccination as the vaccine on which they focused their efforts as a champion. Furthermore, PCPs who worked with champions reported more positive HPV vaccine recommendation practices and perceptions of their clinic’s HPV vaccination rates. Taken together, these findings suggest that champions may be effective at increasing HPV vaccination, although our study’s cross-sectional design and reliance on self-reported data preclude our ability to establish causality. In prior research, several quasi-experimental and observational studies have identified positive associations between vaccine champions and influenza vaccination, the use of vaccine reminder and recall messages in pediatric and public clinics, and the presence of standing order programs in primary care [22–24]. In contrast, several cluster-randomized trials assessing multimodal interventions, including the designation of a champion, found no or modest effects on several vaccines in obstetrics and gynecology clinics, but these studies were not designed to evaluate the impact of champions specifically [25–27]. Thus, while vaccine champions are a highly promising implementation strategy, further randomized studies will be needed to provide higher quality evidence of their effectiveness for changing their colleagues’ practices and perceptions and improving HPV vaccination rates.

Towards that end, our findings provide several points of guidance for researchers and quality improvement leaders who seek to engage vaccine champions. First, our finding that champions are highly prevalent suggests that interventionists can expect to consistently find champions in adolescent primary care, although more targeted efforts to identify them may be needed in lower-volume practices that are not part of healthcare systems or that are located in the South or West, where champions were less common. Second, we found that champions came from diverse backgrounds in terms of training, which suggests that interventionists should consider physicians, advanced practice providers, and nurses in the champion role. In fact, given the diversity in PCPs’ relationships to champions, multidisciplinary teams of champions may be the ideal. Such an approach would be consistent with prior studies which have found that engaging multiple champions is preferable to having champions serve alone and could also offer potential relief for over-burdened physicians with limited time for additional duties [2]. Finally, when asked to identify their closest champion, PCPs were equally likely to identify a colleague who was or was not recognized for being a champion in their professional title or formal job description. For this reason, interventionists should consider both institutionally-recognized champions as well as champions who may take on the role more informally, based on their own interest and dedication.

Strengths of this study include data from a large, national sample of PCPs with multidisciplinary representation across physicians, advanced practice providers, and nursing staff in adolescent primary care. Our cross-sectional study design allowed us to collect novel data on champions’ attributes and strategies, but also constitutes a limitation insofar as we cannot establish whether associations, such as that between knowing a champion and positive HPV vaccine recommendations, are causal in nature. Another limitation to our study is the challenge of defining a vaccine champion to PCPs working in adolescent primary care, a field in which support for vaccination services is the norm. We conducted extensive cognitive testing to define vaccine champions as those who help their colleagues improve vaccination rates, as opposed to more general promotion of vaccines to patients and their families. Nevertheless, this concept is vulnerable to misinterpretation, which could lead to overestimation of champion prevalence. Similarly, though we asked PCPs about various champion implementation strategies and whether champions led quality improvement projects, it is possible these champions contribute in various ways or undertake strategies not captured by our survey. Finally, we note that our findings are based on PCPs’ perceptions and self-report. Results describing PCP’s outlook on their performance and the performance of their clinics are subject to biases, including social desirability, but are nonetheless valuable in providing data to inform future intervention research to establish the champions’ impact on vaccination rates.

## Conclusion

While the implementation science literature frequently invokes champions, studies directly assessing their role in improving clinical outcomes like vaccination are scarce. Champions are highlighted for their potential to successfully implement clinic-based interventions, but overcoming status quo and other organizational resistance are inherently challenging, and a more detailed understanding of champions will better inform efforts to deliver and sustain health services. To this end, our study finds that vaccine champions are widespread but underutilized in quality improvement projects in adolescent primary care, include PCPs of various training backgrounds, and may or may not have a formal title. The relatively low proportion of champions who participate in quality improvement efforts may indicate the need for training and support for champions to lead more formal initiatives. Future research should explore barriers and facilitators to champions’ work in guiding implementation of health services and promoting adolescent vaccines. Importantly, we find an intriguing association between working with a champion and more positive HPV vaccination behaviors and perceptions, which warrant further evaluation in randomized controlled trials.

### Supplementary Information

**Additional file 1: Supplemental Table 1.** Survey items

**Additional file 2. Supplementary Table 2.** Characteristics of PCPs in our sample versus those in the Current Population Survey.

## Data Availability

The datasets generated and/or analyzed during the current study are available upon request upon study completion.

## Abbreviations

ERIC: Expert recommendations for implementing change
HPV: Human papillomavirus
PCP: Primary care professional
US: United states
