# Supplementary material for: Who are vaccine champions and what implementation strategies do they use to improve adolescent HPV vaccination? Findings from a national survey of primary care professionals
Source: Implement Sci Commun. 2024 Mar 22;5:28. doi: 10.1186/s43058-024-00557-0 (PMC10958944; doi:10.1186/s43058-024-00557-0)
Supplement: Supplementary file 1 — Additional file 1: Supplemental Table 1. Survey items [file 43058_2024_557_MOESM1_ESM.docx]

**Supplemental Table 1.** Survey items

| **SCREENER** |
| --- |
| X02 Training  What is your medical training?  1=Physician (MD, DO)  2=Physician assistant (PA)  3=Advanced practice nurse (nurse practitioner/NP, advanced practice nurse/APN, clinical nurse specialist/CNS)  4=Registered nurse (RN)  5=Licensed practical or vocational nurse (LPN, LVN)  6=Certified nursing assistant (CNA)  7=Medical assistant (MA) |
| X09.1 Specialty  [If X02=1]  What is your primary specialty? |
| X09.2 Specialty  [If X02 ne 1]  What is the specialty of your clinic or practice?  (Check all that apply.)  If you work at multiple clinics or practices, answer about the one where you spend the most time on primary care for children ages 9-12. |
| X11 HPV vaccination  Do you have a role in HPV vaccination for children ages 9-12?  Say yes if you do any of these:  -Assess children’s vaccination status  -Let parents know children are due for the vaccine  -Recommend the vaccine  -Address parents’ questions and concerns about the vaccine  -Administer the vaccine |
| X08.1 clinic location  Where is your clinic or practice located?  If you work at multiple clinics or practices, answer about the one where you spend the most time on vaccination for children ages 9-12.  State is needed to continue with the survey.  [State drop down menu]  1-51=States with DC |
| X08.2 clinic location  Where is your clinic or practice located?  If you work at multiple clinics or practices, answer about the one where you spend the most time on vaccination for children ages 9-12.  County is needed to continue with the survey.  [County drop down menu]  1-999=Counties within state |

| **SURVEY** |
| --- |
| X06 Vaccination roles  What role do you have in HPV vaccination for children ages 9-12?  (Check all that apply.)  1=Assess children’s vaccination status  2=Let parents know children are due for the vaccine  3=Recommend the vaccine  4=Address parents’ questions and concerns about the vaccine  5=Administer the vaccine |
| J01 Prompt  Some health care professionals are known for **helping their colleagues improve vaccination rates**. They are passionate about sharing vaccine-related information, data, tools, and encouragement with others in their clinic.  We will call them **vaccine champions**. |
| J02 Prevalence  How many vaccine champions do you currently work with?    Consider anyone who goes **above and beyond** to help you or others in your clinic improve vaccination rates.  You can count physicians, nursing staff, administrators, quality improvement staff, and others.    **Do not count yourself.**    **[If response=1, skip to K10]**  1=0 champions  2=1 champion  3=2 champions  4=3 champions  5=4 champions  6=5 champions  7=6 champions  8=7 champions  9=8 or more champions |
| K01 Prompt  You indicated that you work with {#} vaccine [champion/champions]. For the next questions, think about {**that person/the one you work with most closely**}.  Do not answer about yourself. |
| K02 Activities and character.  What is the vaccine champion’s training?  (*Select one.)*    1=Physician  2=Physician assistant  3=Advanced practice nurse  4=Nurse  5=Certified nursing assistant or medical assistant  99=None of these |
| K03 Activities and character.  What role does the vaccine champion play in your clinic?  (Check all that apply.)  1=Patient care team member  2=Vaccine stock manager  3=Clinic manager  4=Other administrator  5=Quality improvement coordinator  99=None of these |
| K04 Activities and character.  Which strategies does the vaccine champion use to improve vaccination rates?  (Check all that apply.)  1=Shares information with colleagues  2=Encourages colleagues to improve  3=Shares data on vaccination rates  4=Leads quality improvement projects  5=Communicates effectively with patients and their families  99=None of these |
| K05 Activities and character.  Which vaccination rates does the vaccine champion work to improve?  (Check all that apply.)  1=HPV  2=Seasonal influenza  3=Covid-19  4=Other pediatric vaccinations  5=Other adult vaccinations  6=All of the above |
| K06 Activities and character.  How is the vaccine champion’s role in your clinic recognized?    Is being a vaccine champion…  (Check all that apply.)  1=One of their job titles  2=Part of their formal job description  3=Something they do outside of their title or formal role |
| K07 Activities and character.  Which qualities best describe the vaccine champion?  (Check all that apply.)  1=Knowledgeable about vaccines  2=Knowledgeable about your clinic  3=Highly respected by colleagues  4=Trusted by patients and their families  5=Effective communicator  99=None of these |
| K08 Activities and character.  How effective is the vaccine champion at improving vaccination rates in your clinic?  1=Not at all effective  2=Slightly effective  3=Moderately effective  4=Very effective  5=Extremely effective |
| K09 Activities and character.  How closely do you work with the vaccine champion?  1=Not at all closely  2=Slightly closely  3=Moderately closely  4=Very closely  5=Extremely closely |
| Z07.1  **[If recommends HPV vaccine (X06=3)]**  Do you routinely recommend HPV vaccine for patients starting at…  1=Ages 9-10  2=Ages 11-12  3=Ages 13-17  4=An older age  99=You don’t routinely recommend HPV vaccine |
| Z09 HPV vaccination rate  In {STATE}**,** about {X}% of children ages 13 to 17 are up to date on HPV vaccination. Do you estimate your clinic’s HPV vaccination rate to be...  **[STATE piped in from X08.1 and X piped in from state-specific rate data provided]**  1=At or above {X}%  2=Below {X}% |
| Z03  Practice type  Which of the following best describes your clinic?  1=Solo practice  2=Group practice  3=Hospital or academic institution  4=Federally qualified health center or community health center  5=State or local department of public health  6=Local, community or non-profit organization  7=Other |
| Z02  Integrated healthcare system  Is your clinic part of a healthcare system or network?  0=No  1=Yes, a system of 1-4 clinics  2=Yes, a system of 5 or more clinics |
| Z12  Patient load  About how many children ages 9-12 do you see in a typical week?  1=0 children  2=1-9 children  3=10-24 children  4=25-49 children  5=50 or more children |
| Z15  Additional clinics  Aside from your primary clinic, do you have a role in HPV vaccination for children ages 9-12 at other clinics?  0=No  1=1 other clinic  2=2 other clinics  3=3 or more other clinics |
| Z15.1 (state 2)  Z15.2 (county 2)  Z15.3-Z15.8  Rurality of additional clinic  **[If Z15=1]**  Where is your other clinic located?  **[If yes to Z15 > 1]**  Where are your other clinics located?  [**State drop down menu]**  1-51=States with DC  **[County drop down menu]**  1-999=Counties within state  **[Provide up to 3 state and county options depending on answer to Z15]** |
| X03 Years in practice  How many years have you been a {fill response from X02 Training}?  _________ years  **[Record continuous variable; allow values from 0 – 70]**  Count years only after residency, fellowship, or initial required training. |
| X04 Gender  Select the option that best describes your gender.  1=Woman  2=Man  3=Nonbinary  4=Another gender  5=Prefer not to say |
| X05 Race/ Ethnicity  What is your race or ethnicity?  (Check all that apply.)  1=White  2=Hispanic, Latino, or Spanish  3=Black or African American  4=Asian  5=American Indian or Alaska Native  6=Middle Eastern or North African  7=Native Hawaiian or Other Pacific Islander  8=Some other race or ethnicity (please specify: _____)  9=Prefer not to say |
| X15 Age  How old are you?  _______ years  **[Record continuous variable, allow values from 18-99]** |
