# Supplementary material for: Who are vaccine champions and what implementation strategies do they use to improve adolescent HPV vaccination? Findings from a national survey of primary care professionals
Source: Implement Sci Commun. 2024 Mar 22;5:28. doi: 10.1186/s43058-024-00557-0 (PMC10958944; doi:10.1186/s43058-024-00557-0)
Supplement: Supplementary file 2 — Additional file 2. Supplementary Table 2. Characteristics of PCPs in our sample versus those in the Current Population Survey. [file 43058_2024_557_MOESM2_ESM.docx]

| **Supplementary Table 2.** Characteristics of PCPs in our sample versus those in the Current Population Survey | | | | | | |
| --- | --- | --- | --- | --- | --- | --- |
|  | White | | Female | | Mean age (years) | |
|  | Sample | CPS | Sample | CPS | Sample | CPS |
| Physicians | 62% | 69% | 55% | 43% | 47 | 53 |
| Advanced practitioners^1^ | 76%C | 85% | 84% | 92% | 43 | 43 |
| Registered nurses | 78% | 68% | 89% | 98% | 46 | 48 |
| Nursing staff^2^ | 70% | 53% | 91% | 89% | 43 | 40 |
| *Notes.* CPS: 2022 Current Population Survey https://www.census.gov/programs-surveys/cps/technical- documentation/questionnaires.html  ^1^ Includes physician assistants and nurse practitioners  ^2^ Includes CNAs, MAs, LVNs, LPNs | | | | | | |
